# Development of Clinical-Stage Human Monoclonal Antibodies That Treat Advanced Ebola Virus Disease in Nonhuman Primates

**DOI:** 10.1093/infdis/jiy285

**Published:** 2018-05-31

**Authors:** Kristen E Pascal, Drew Dudgeon, John C Trefry, Manu Anantpadma, Yasuteru Sakurai, Charles D Murin, Hannah L Turner, Jeanette Fairhurst, Marcela Torres, Ashique Rafique, Ying Yan, Ashok Badithe, Kevin Yu, Terra Potocky, Sandra L Bixler, Taylor B Chance, William D Pratt, Franco D Rossi, Joshua D Shamblin, Suzanne E Wollen, Justine M Zelko, Ricardo Carrion, Gabriella Worwa, Hilary M Staples, Darya Burakov, Robert Babb, Gang Chen, Joel Martin, Tammy T Huang, Karl Erlandson, Melissa S Willis, Kimberly Armstrong, Thomas M Dreier, Andrew B Ward, Robert A Davey, Margaret L M Pitt, Leah Lipsich, Peter Mason, William Olson, Neil Stahl, Christos A Kyratsous

**Affiliations:** 1 Regeneron Pharmaceuticals, Inc., Tarrytown, New York; 2 Virology Division, US Army Medical Research Institute of Infectious Diseases, Ft. Detrick, Maryland; 3 Pathology Division, US Army Medical Research Institute of Infectious Diseases, Ft. Detrick, Maryland; 4 Center for Aerobiological Sciences, US Army Medical Research Institute of Infectious Diseases, Ft. Detrick, Maryland; 5 Office of the Commander, US Army Medical Research Institute of Infectious Diseases, Ft. Detrick, Maryland; 6 Department of Virology and Immunology, Texas Biomedical Research Institute, San Antonio; 7 Department of Integrative Structural and Computational Biology, The Scripps Research Institute, La Jolla, California; 8 Biomedical Advanced Research and Development Authority, Office of the Assistant Secretary for Preparedness and Response, US Department of Health and Human Services, Washington, DC

**Keywords:** EBOV, filovirus, monoclonal antibodies, treatment

## Abstract

**Background:**

For most classes of drugs, rapid development of therapeutics to treat emerging infections is challenged by the timelines needed to identify compounds with the desired efficacy, safety, and pharmacokinetic profiles. Fully human monoclonal antibodies (mAbs) provide an attractive method to overcome many of these hurdles to rapidly produce therapeutics for emerging diseases.

**Methods:**

In this study, we deployed a platform to generate, test, and develop fully human antibodies to *Zaire ebolavirus*. We obtained specific anti-Ebola virus (EBOV) antibodies by immunizing VelocImmune mice that use human immunoglobulin variable regions in their humoral responses.

**Results:**

Of the antibody clones isolated, 3 were selected as best at neutralizing EBOV and triggering FcγRIIIa. Binding studies and negative-stain electron microscopy revealed that the 3 selected antibodies bind to non-overlapping epitopes, including a potentially new protective epitope not targeted by other antibody-based treatments. When combined, a single dose of a cocktail of the 3 antibodies protected nonhuman primates (NHPs) from EBOV disease even after disease symptoms were apparent.

**Conclusions:**

This antibody cocktail provides complementary mechanisms of actions, incorporates novel specificities, and demonstrates high-level postexposure protection from lethal EBOV disease in NHPs. It is now undergoing testing in normal healthy volunteers in preparation for potential use in future Ebola epidemics.

The 2013–2016 Ebola virus pandemic in West Africa resulted in more than 28000 cases and ~11000 deaths throughout Guinea, Sierra Leone, and Liberia (http://www.who.int/csr/disease/ebola/en/). Despite being ultimately controlled, *Zaire ebolavirus* (EBOV) and other single-stranded ribonucleic acid viruses in the *Filoviridae* family, continue to pose a high risk for causing epidemics with high mortality rates [1]. In addition to the mortality associated with acute infections and disease, sequelae of EBOV infections include neurological and ocular complications, joint and muscle pain, and virus persistence for many months [2, 3]. This persistence is particularly concerning from the public health perspective given that most of the population is still immune naive. Currently, there are no approved therapies or vaccines to treat or prevent infections by EBOV or other filoviruses, necessitating development of clinical-grade therapeutics.

The main target of EBOV vaccines and therapeutic antibodies is the virus glycoprotein (GP), which is currently the only validated therapeutic target on the virion surface. Glycoprotein is produced from an alternative transcript of the GP gene, with soluble GP (sGP) being the main product [4]. Glycoprotein is a class I fusion protein that is posttranslationally cleaved by furin into 2 disulphide-linked subunits (GP1 and GP2) that form a trimer of heterodimers on the surface of the virion and infected cells [5, 6]. Once host cell-bound virions are internalized into endosomes, the viruses are exposed to low pH, and further proteolytic cleavage of GP reveals GP1 surfaces that bind the endosomal receptor Niemann-Pick C1 (NPC1), which is required for penetration of the nucleocapsid into the cell cytoplasm [7] and initiation of replication. The sGPs can be readily detected in the sera of infected animals, and patients and plays a role in pathogenesis [8].

Multiple lines of evidence support GP as an effective therapeutic target. Live-attenuated recombinant vesicular stomatitis virus (rVSV) vaccine decorated with EBOV GP protects nonhuman primates (NHPs) from EBOV challenge [9]. Protection induced by this rVSV vaccine correlated with development of GP-targeting antibodies [10]. The vaccine was used in a ring vaccination, open-label, cluster-randomized trial during the 2013–2016 pandemic and showed evidence of efficacy and safety in preventing EBOV disease when administered to primary and secondary contacts of infected patients during an outbreak [11]. In addition, GP-specific antibodies isolated from survivors of EBOV infection or immunized animals have been shown to effectively treat EBOV disease in infected NHPs [12–14]. However, efficacious therapeutic antibody preparations are likely to require the combination of neutralizing and nonneutralizing antibodies with multiple mechanisms of action, including antibody effector functions [15].

The risk of new filovirus epidemics calls for development and production of therapeutics that can be rapidly deployed. To date, EBOV antibody therapies have been developed either by humanization of rodent-generated antibodies or by isolation of antibody genes from infected patients. Each method has significant drawbacks: antibody therapeutics have also faced supply constraints due to challenges with production methods [16], requirements for sequence reverse engineering [17], and/or lack of manufacturing-ready cell lines, thereby preventing large-scale production and controlled testing for treatment of EBOV disease. In this study, we describe the use of a platform to generate, select, test, and manufacture a panel of fully human, noncross-competing monoclonal antibodies (mAbs) to treat EBOV disease. Mice encoding fully human antibody variable region gene segments (VelocImmune mice) [18, 19] were immunized, and 3 EBOV GP-binding antibodies with complementary biological properties were identified. Chinese hamster ovary (CHO) isogenic cell banks expressing each of these mAbs individually were made, and mAbs were immediately manufactured. When used as a cocktail, the 3 mAbs reversed severe disease symptoms and greatly promoted survival of rhesus macaques when administered at 5 days post-EBOV challenge, late in the disease course in this animal model. In this study, we describe the biological properties of the antibodies, where they bind to GP, and the outcomes of testing in the NHP model of disease.

## METHODS

### Viruses and Cell Lines

293T cells (human embryonic kidney [HEK] cell line, obtained from American Type Culture Collection [ATCC]) and Huh-7 cells (human hepatocarcinoma cell line, obtained from JCRB Cell Bank) were grown in high glucose Dulbecco’s modified Eagle’s media ([DMEM] Gibco) containing 10% v/v fetal bovine serum ([FBS] Gibco), 1% l-glutamine, and antibiotics (penicillin/streptomycin; Gibco) at 37°C in a 5% CO_2_ atmosphere. Vero E6 (monkey kidney epithelial cells, obtained from ATCC) were grown in minimal essential media (Corning) supplemented with 10% v/v FBS (Sigma Aldrich), 1% l-glutamine (Gibco), and 1% penicillin/streptomycin (Gemini Bio Products) at 37°C in a 5% CO_2_ atmosphere. The EESYR CHO cell line is derived from CHO cells (CHO cell line, obtained from ATCC) and has been previously described (US Patent 7771997). Transfections were performed using Lipofectamine LTX and Plus Reagent (Life Technologies) following the manufacturer’s instructions.

The Makona strain of EBOV was originally isolated by Dr. Stephan Gunther [20] and was obtained with permission through Dr. Gary Kobinger (Special Pathogens Program, National Microbiology Laboratory, Public Health Agency of Canada, Winnipeg, Manitoba). The Mayinga strain of EBOV was obtained from Dr. Heinrich Feldmann (Laboratory of Virology, National Institute of Allergy and Infectious Diseases [NIAID], National Institutes of Health, Rocky Mountain Laboratories, Hamilton, MT).

The EBOV kikwit-9510621 was used for animal challenges at Texas Biomedical Research Institute. A second cell-culture passage (P2) of EBOV kikwit-9510621 was obtained from Dr. Tom Ksiazek (at NIAID’s World Reference Center for Emerging Viruses and Arboviruses at University of Texas Medical Branch’s Health Galveston National Laboratory) in 2012 and propagated at Texas Biomedical Research Institute. The stock virus was passaged for a third time in Vero E6 cells and had a titer of 2.1 × 10^5^ plaque-forming units (pfu)/mL. The challenge stock has been confirmed to be wild-type EBOV by deep sequencing and was attributed the lot no. 201209171. Sequencing showed that the abundance of the genomes possess 7U at the editing site locus is 94.3%

Guinea pig-adapted EBOV master originated from human serum specimen (057931) that was passaged as previously described [21]. A stock of the virus was kindly provided by Dr. Heinrich Feldman. Stocks were used to infect Vero E6 cells and incubated until a 3+ cytopathic effect was observed, and then the supernatant was removed, clarified, and frozen in aliquots. Virus stocks were counted by conventional plaque titration.

### Nucleic Acids

Codon-optimized complementary deoxyribonucleic acid (DNA) sequence encoding for EBOV GP (Makona strain; GenBank no. KJ660346) was synthesized and cloned into expression vectors using standard methods. psPAX2 and pWPXLd-GFP encoding for human immunodeficiency virus (HIV) gag pol and green fluorescent protein in the context of an HIV genome were obtained from the Tronolab (Ecole Polytechnique Fédérale de Lausanne, Switzerland). pCK-retro-luc was described before [22].

### Proteins

Soluble EBOV GP (Makona strain) was purchased from Sino Biological.

### Generation and Expression of Anti-Ebola Virus Glycoprotein Antibodies

VelocImmune mice [18, 19] comprising DNA encoding human immunoglobulin (Ig) heavy and kappa light chain variable regions and mouse constant regions were immunized with DNA expression EBOV GP and purified EBOV GP protein as immunogens using standard techniques. When the desired immune response was achieved, splenocytes were harvested.

Splenocytes fusion and isolation of EBOV GP-reactive B cells were performed using previously described methods [22]. The most potent antibodies for binding, neutralization, and FcγRIIIa signaling were selected for subsequent analysis.

Recombinant anti-EBOV GP antibodies were produced in CHO cells after stable transfection with paired expression plasmids containing heavy chain and light chain derived from the same B cell. Antibodies were purified from culture supernatants by Protein A affinity chromatography. Clinical-grade antibody was further processed via nonaffinity column chromatography and dedicated virus removal/inactivation steps.

### Binding of Antibodies to Purified EBOV-GP Protein

Equilibrium dissociation constants (*K*_D_ values) for the interaction of anti-EBOV GP antibodies and recombinant EBOV GP protein were determined via surface plasmon resonance (SPR) using a Biacore 4000 instrument (GE Healthcare). Specific Biacore kinetic sensorgrams were obtained by the standard double-referencing procedure. The kinetic parameters were obtained by globally fitting the data to a 1:1 binding model using Biacore 4000 Evaluation curve-fitting software. The dissociation rate constant (*k*_d_) was determined by fitting the change in the binding response during the dissociation phase, and the association rate constant (*k*_a_) was determined by fitting EBOV GP binding at different concentrations. The *K*_D_ was calculated from the ratio of the *k*_d_ and *k*_a_. The dissociative half-life (*t*_1/2_) was calculated as ln2/*k*_d_.

### Binding of Antibodies to Purified Soluble EBOV-sGP Protein

Binding of anti-EBOV GP antibodies to soluble EBOV-sGP was determined using a real-time, label-free biolayer interferometry assay (BLI) Octet HTX system at 25°C in HBS-EP Octet buffer (10 mM Hepes, 150 mM NaCl, 3.4 mM ethylenediaminetetraacetic acid [EDTA], and 0.05% [vol/vol] surfactant Tween-20, pH 7.4, 1 mg/mL bovine serum albumin [BSA]) with the plate shaking at 1000 rpm. Data analysis was performed by using ForteBio Data Analysis software version 9.0.

### Binding Competition Assay

Epitope binning between anti-EBOV GP antibodies was determined using the Octet HTX system equipped with anti-His sensors (Pall ForteBio). Experiments were performed at 25°C in HBS-EP Octet buffer with the plate shaking at 1000 rpm. Data analysis was performed by using ForteBio Data Analysis software, version 9.0.

### Sequential Binding of REGN3470, REGN3471, and REGN3479 to EBOV GP.10xhis by Surface Plasmon Resonance-Biacore

The EBOV GP.10xhis was captured onto Biacore amine coupled anti-His CM5 sensor surfaces. Next, 50 μg/mL of the first mAb was injected for 5 minutes to reach binding saturation followed by injection of the second mAb and third mAb under the same conditions. The binding sensorgrams were referenced to a flow cell with no EBOV GP protein captured. All curves were zeroed before each injection, and the relative binding levels were determined after binding saturation was achieved. This was repeated for anti-EBOV GP antibodies and the isotype control antibody (REGN1932) at all sequence permutations. The real-time binding response was monitored during the course of the experiment, and the binding response at the end of every step was measured. Data were analyzed using T200 evaluation software, version 2.0.

### Negative Stain Electron Microscopy

Antibodies were digested using 4% w/w papain for 5 hours. The fragment antigen-binding agents (Fab) were purified using a Protein A column (GE Healthcare) followed by size exclusion chromatography with an S200 Increase (S200I) column (GE Healthcare). The Fabs were added to mucin-like, domain-containing, transmembrane-deleted Ebola GP (EBOV GPΔTM) in 10 molar excess and allowed to incubate overnight at 4°C. Complexes were subsequently purified using an S200I column. Ebola GP and Fab complexes were deposited on thin carbon-coated, 400-mesh Cu grids and stained with 2% (w/v) uranyl formate. Images were collected automatically using Leginon [23] with Tietz TemCam-F416 CMOS camera on a Tecnai T12 Spirit electron microscope operating at 120 keV. The magnification was 52000 at the specimen plane, and a constant 1.5-micron defocus was applied. Images were processed in Appion [24]. Particles were selected using DogPicker [25], placed in stacks [26], and sorted into reference-free 2-dimensional (2D) classes averages by multivariate statistical analysis/multireference alignment [27]. After removing abhorrent particles, resulting particle substacks were submitted to iterative stable alignment and clustering to generate a final set of 2D class averages [28]. Individual class averages were used to generate an initial 3D model using EMAN2 [29], which were then refined against raw particles, enforcing C3 symmetry, to general the final 3D model. Model images and manipulation were performed in UCSF Chimera [30]. Maps were segmented into Fab and core GP components using Segger. All maps were aligned to a single GP density generated from the c13C6/c4G7 GPΔTM map [31] to generate the 3 Fab models of REGN3470, 3471, and 3479.

### Generation of Pseudotyped Viruses

Retrovirus particles pseudotyped with EBOV GP protein were generated by cotransfecting 293T cells with a mix of plasmids encoding EBOV GP, psPAX2 encoding virus capside proteins, and pCK-retro-luc encoding a packageable firefly luciferase reporter gene under control of the retrovirus long terminal repeat promoter. Supernatants were harvested 48 hours posttransfection, clarified using centrifugation, concentrated using Lenti-X Concentrator (Clontech), aliquoted, and frozen at −80°C. Control pseudoparticles were generated by substituting the plasmid expressing EBOV GP with a plasmid encoding for vesicular stomatitis virus GP (VSV-G).

### Neutralization Assays

#### Pseudoparticle-Based Assay

The EBOV Zaire 2014 virus-like particles were tested in neutralization assays with Huh7 cells. Serial dilutions of antibody (67 nM–1 pM) were incubated with EBOV GP-pseudotyped virus for 1 hour at room temperature (RT) and added to Huh7 cells. In brief, Huh7 cells were detached using 0.02 M EDTA, washed, and incubated with the antibody/pseudotyped virus mixtures for 72 hours. Infection efficiency was quantitated by luciferase detection with the BrightGlo luciferase assay (Promega, San Luis Obispo, CA), and luminescence (relative luminescence units) was quantified in a Victor X3 plate reader (PerkinElmer, Waltham, MA).

#### Virus-Based Assay

REGN3470, REGN3471, REGN3479, and KZ52 antibodies were analyzed for the ability to neutralize infectious EBOV in Vero cells. Vero cells were plated on 384-well plates in DMEM-10% FBS and allowed to grow to approximately 75% confluence at 37°C. The concentration of each antibody ranged from 25 to 0.006 µg/mL. Plates were transferred to a biosafety level 4 (BSL-4) laboratory to complete the infection assay. The EBOV strains (Mayinga, Kikwit, Makona, and guinea pig-adapted Mayinga) were thawed and diluted. An equivalent volume of infectious EBOV was added to wells (multiplicity of infection between 0.01 and 0.1), and plates were incubated at 37°C for 24 hours. After the incubation period, plates were removed from incubator and inactivated by immersing in 10% neutral-buffered formalin, placed in a heat-sealed bag, and stored at 4°C overnight in the BSL-4 laboratory. Plates were washed 3 times in 1× phosphate-buffered saline (PBS), and cells were permeabilized at RT with 25 µL 0.1% Triton X-100 in 1× PBS for 15–20 minutes. The detergent containing buffer was discarded, and the plates were blocked with 3.5% BSA in PBS for 1 hour at RT. Plates were treated overnight at 4°C with anti-EBOV GP primary antibody (4F3 anti-EBOV GP Antibody; IBT Biosciences) diluted 1:1500 in PBS. Plates were washed twice in PBS for 10–15 minutes. Cells were incubated for 1 hour with Alexa-fluor-488 conjugated antimouse secondary antibody. Secondary antibody was discarded, and plates were again washed twice in PBS for 10–15 minutes. Plates were incubated with 25 µL/well of Hoechst dye (1:50000 in 1× PBS) for 30 minutes at RT. Plates were imaged by fluorescence microscopy. Images were analyzed by Cell Profiler using a HTS pipeline (available upon request). The rate of infection was determined by dividing the number of infected cells with the total number of cells. Infection rates were normalized to the average untreated cell infection rate, and normalized infection rates (relative infectivity) were expressed as percentage of infectivity versus antibody concentration and analyzed in GraphPad Prism software by nonlinear regression analysis. Plaque reduction neutralization (PRNT) 50 and PRNT 80 were determined manually from the graph for each antibody with a sigmoidal curve that showed 50% and 80% inhibition, respectively.

### FcγRIIIa Signaling Assay

The potential of each antibody to induce antibody-dependent cellular cytotoxicity (ADCC) was assessed by activation of FcγRIIIa-mediated nuclear factor of activated T-cell (NFAT) signaling in the presence of EBOV GP expressing target cells. Each anti-EBOV GP mAb or isotype control antibody was incubated with Jurkat effector cells (obtained from ATCC) engineered to express NFAT-Luc and FcγRIIIa ^176^Val (Jurkat/NFAT-Luc/FcγRIIIa ^176^Val) in the presence of HEK293 cells engineered with tetracycline-inducible EBOV GP expression (HEK293/Tet-on/EBOV GP) or, as a negative control, HEK293 cells engineered with the tetracycline transactivator protein alone (HEK293/Tet-on). Jurkat/NFAT-Luc/FcγRIIIa ^176^Val effector cells and HEK293/Tet-on/EBOV GP or HEK293/Tet-on target cells were plated at a 1:1 ratio (30000 cells each) in a 96-well plate. To generate a dose-response curve, individual anti-EBOV GP mAbs, mAb cocktail, or IgG1 isotype control antibody were added to the cells at a concentration range from 0.78 pM to 40 nM, except for REGN3471, which was evaluated at a concentration range of 8.5 pM–500 nM. Plates were incubated at 37°C, 5.0% CO_2_ for 5.5 hours, followed by equilibration at RT for 20 minutes. An equal volume of ONE-Glo luciferase substrate was added to each well, and the plate was incubated at RT for 5 minutes. Relative luminescence units were measured on a Victor X5 multilabel plate reader, and the values were analyzed by a 4-parameter logistic model with an 11-point dose-response curve (GraphPad Prism).

## Ethics Statement

All animal studies were conducted under the protocols approved by the appropriate institutional animal care and use committees (IACUCs) (US Army Medical Research Institute of Infectious Diseases IACUC committee and Texas Biomedical Research Institute [TBRI] IACUC committee). Animal research at the US Army Medical Research Institute of Infectious Diseases (USAMRIID) was conducted in compliance with the Animal Welfare Act, Public Health Service Policy, and other Federal statutes and regulations relating to animals and experiments involving animals. The TBRI protocols adhere to the US Department of Agriculture Animal Welfare regulations, the Public Health Service Policy on Humane Care and Use of Laboratory Animals, the National Research Council Guide for the Care and Use of Laboratory Animals, and the policies established by TBRI.

### Nonhuman Primate Welfare

Routine feeding occurred and environment enrichment provided as per established standard operation procedures. Animals were singly housed to properly assess signs of disease, and this allowed for more accurate determination of food consumption, water intake, and fecal and urine output, which are necessary in determining morbidity. No analgesics for clinical signs of disease were used because the use of analgesics may have a secondary effect of masking clinical signs of infection that would prevent evaluation of disease severity. In addition, other analgesics may exacerbate clotting issues. There may also be secondary unintended consequences with interactions between the candidate drug and the analgesic. Euthanasia criteria was created to help minimize the pain and distress the animals experience while still allowing time to collect sufficient data to validate the treatment regimen. Euthanasia was humanely performed when animals met established moribund criteria and was in accordance with approved methods under the American Veterinary Medical Association Guidelines for the Euthanasia of Animals. The USAMRIID Protocols for housing, feeding, and environmental enrichment and steps to minimize suffering of NHPs adhere to principles stated in the Guide for the Care and Use of Laboratory Animals, National Research Council, 2011.

## In Vivo Studies

All animal studies were performed in a BSL-4 laboratory at TBRI or USAMRIID. Veterinary staff were blinded to treatment status of all animals until the end of studies.

### Guinea Pig Study (Texas Biomedical Research Institute)

For the efficacy study of single antibody treatment, male guinea pigs (Hartley strain, 5 to 6 weeks old) were obtained from Charles River Laboratories (Wilmington, MA) and randomly assigned to treatment groups with 6 animals per group. They were placed in the BSL-4 laboratory 1 week before virus challenge and fed and cared for daily. All of the animals were challenged intraperitoneally with a target dose of 1000 pfu of guinea pig-adapted EBOV (originally provided by Dr. Heinrich Feldman, Rocky Mountain Laboratories, National Institutes of Health, Hamilton, MT). After 1 day, each group was treated by intraperitoneal dosing with 5 mg of antibody per animal or an equivalent volume of saline for the placebo control. All animals were observed at least twice daily for weight loss and clinical signs, and surviving animals were sacrificed 4 weeks after virus challenge. Blood samples were collected 4 days after virus challenge, and sera were isolated and stored at −80^o^C.

### Nonhuman Primate Study (US Army Medical Research Institute of Infectious Diseases)

Research was conducted under an IACUC-approved protocol in compliance with the Animal Welfare Act, Public Health Service Policy, and other Federal statutes and regulations relating to animals and experiments involving animals. The facility where this research was conducted is accredited by the Association for Assessment and Accreditation of Laboratory Animal Care, International and adheres to principles stated in the Guide for the Care and Use of Laboratory Animals, National Research Council, 2011. Research-naive, adult rhesus macaques (*Macaca mulatta*) of Asian origin, mixed male and female, were obtained from the USAMRIID colony. Each animal was screened and found negative for filovirus antibodies. Macaques were equally balanced by sex and weight then randomized and coded for blinding. All animals were challenged intramuscularly in the caudal thigh with a target dose of 1000 pfu EBOV (Kikwit, 7U). Phlebotomies were conducted via saphenous venipuncture on the following days: prechallenge, 0; day of challenge, 3, 5, 8, 11, 14, 21, 28, and terminal. Antibody treatments (50 mg/kg) were administered in a single bolus of less than 30 mL over approximately 60 seconds through a 7F Groshong central venous catheter (Bard Access Systems, Salt Lake City, UT) or via 21- to 25-gauge winged infusion needle (Terumo Medical Products, Somerset, NJ). At each phlebotomy and/or treatment, animals were lightly anesthetized with ketamine (Vedco, St. Joseph, MO). Nonhuman primates were observed approximately every 4–8 hours when showing signs of disease.

#### Telemetry

Real-time telemetry analysis was performed as detailed previous [32]. In brief, NHPs were implanted with radio telemetry devices (T2J; Konigsberg Instruments, Pasadena, CA) to monitor their temperature and activity throughout the experiment. Data were acquired and analyzed via Notocord-hem Evolution software (version 4.3.0.43; Notocord Inc., Newark, NJ).

#### Viral Titration

Challenge doses were determined via plaque assay on the day of challenge. Vero E6 cells were plated in 6-well plates the day before challenge, and serial dilutions of the challenge material were incubated atop the monolayer for 1 hour at 37°C. After this incubation, the monolayer was overlain with a 0.5% agarose (SeaKem ME Agarose; Lonza, Rockland, ME) solution. After 1 week at 37°C, the cells were again overlain with neutral red and incubated overnight at 37°C. Plaques were counted over a light box the following day. This same process was repeated to enumerate each animal’s viremia; however, instead of challenge material, serum was serially diluted and the plaque assay performed as described above.

#### Serum Chemistry

Serum separator tubes (Greiner Bio-One, Monroe, NC) were inverted after each phlebotomy and left to clot for approximately 30 minutes, at which time they were centrifuged at 1800 relative centrifugal force (rcf) for 10 minutes at RT. Serum was then pipetted into a Piccolo General Chemistry 13 disk and run on a Piccolo Point of Care Analyzer (Abaxis, Union City, CA).

#### Hematology

K3EDTA tubes were inverted by hand and applied to the sampling port of an Advia 120 (Siemens Healthcare, Palvern, PA) to achieve complete blood counts. After running the whole blood on the Advia 120, the sample was centrifuged at 2500 rcf for 10 minutes and the plasma was removed from the cell pellet.

#### Quantitative Reverse Transcription-Polymerase Chain Reaction

The EDTA plasma was mixed with TriReagent LS (Sigma-Aldrich), 100 to 300 µL, respectively. Polymerase chain reaction (PCR) was performed as described previously [32].

### Nonhuman Primate Study (Texas Biomedical Research Institute)

Eighteen male rhesus macaques (*M mulatta*, 3–6 years old) were obtained from Covance (Alice, TX), ranging from 4 to 8 kg, and randomly assigned to treatment groups. They were placed in the BSL-4 laboratory 8 days before virus challenge for acclimation and fed and cared for daily. All of the animals were challenged intramuscularly with a target dose of 1000 pfu of EBOV Kikwit. After 5, 8, and 11 days, each group was treated intravenously with 50 or 150 mg/kg of an antibody cocktail or an equivalent volume of PBS. All animals were observed at least twice daily for weight loss and clinical signs, and surviving animals were euthanized 4 weeks after virus challenge. Blood samples were collected on day 0, 3, 5, 8, 11, 14, 21, and 28 days after virus challenge. Plasma and serum was prepared for biochemistry and viremia analysis and stored at −80^o^C. For quantification of virus loads in animal sera, plaque assays were performed. Vero E6 cells grown to confluence in 6-well plates were inoculated with dilutions of animal serum. After 1-hour incubation, the supernatant was replaced with Eagle’s minimum essential medium containing 0.5% agarose and 2% FBS and incubated at 37^o^C. After 1 week, the same media with addition of 2% neutral red was added to each well. After additional 1-day incubation at 37^o^C, plaques were manually counted.

## RESULTS

### Isolation of Three Antibodies That Simultaneously Bind to Ebola Virus-Glycoprotein With High Affinity

Our goal was to rapidly select a cocktail of 3 noncompeting EBOV GP-specific fully human mAbs that possess diverse biological properties/mechanisms of action. To obtain these, VelocImmune mice were immunized with DNA constructs encoding the GP protein and/or recombinant purified GP protein (Makona strain). To select the best therapeutic antibodies from the pool of GP-specific antibodies obtained from these immunized mice, several in vitro screening assays were used to identify antibodies that collectively provide the following properties: (1) blocking entry of EBOV GP-pseudotyped particles into susceptible cells, (2) ability to remain bound to GP at endosomal pH to prevent interaction with intracellular receptors and membrane fusion [33], (3) lack of cross-competition to ensure that antibodies simultaneously bind to GP, (4) binding to sGP to block its potential role in EBOV pathogenesis, and (5) Fc effector function to mediate killing of EBOV-infected cells by immune cells, which was previously suggested as important for protection [16]. To facilitate discovery of antibodies that mediated this latter activity, our screening was undertaken with antibodies produced in a CHO cell line that had been selected for its ability to produce antibodies with very low levels of terminal fucosylation (data not shown), because low fucosylation has been reported to enhance antibody effector function [34] and contribute to anti-EBOV activity [35]. Using the above screening criteria, 3 mAbs (REGN3470, REGN3471, and REGN3479—all produced in low-fucose cell lines) were selected within 10 weeks of initiating our immunization campaign.

The selected lead antibodies were stably expressed in EESYR CHO cell lines by recombination-mediated cassette exchange into the EESYR locus. Genes recombined into the EESYR locus are exceptionally stable, and precise recombination is monitored by the exchange of fluorescent protein genes in the chromosome and the in-coming targeting vector (US Patent 7771997). Using these cells, isogenic CHO cell banks were established that yielded several grams per liter of mAb in single use bioreactors (data not shown). The purified proteins were available for in vitro testing approximately 2 months after immunization, larger-scale purified lots used for NHP studies were available approximately 2 months later, and good manufacturing practices (GMP)-quality antibodies that were used for some of the in vivo studies and testing for safety in human volunteers were available less than 1 year after initiation of the immunization campaign.

REGN3470, REGN3471, and REGN3479 bind to EBOV GP with high affinity, retain binding at low pH, and do not cross-compete. Surface plasma resonance was used to determine the kinetic binding parameters for the interaction of REGN3470, REGN3471, and REGN3479 with recombinant, histidine-tagged Makona strain EBOV GP ectodomain protein (EBOV GP.10xhis) at 25°C and pH 7.4. Each test antibody was captured onto the sensor chip surface via its Fc region, followed by injection of recombinant EBOV GP. The *K*_D_ ranged from 3 to 8.4 nM, reflecting high-affinity binding for each of the 3 antibodies (Table 1). Additional studies examined whether the antibodies retained binding over the normal range of endosomal pH (5–7.4). As shown in Table 2, the mAbs displayed a less than 2-fold (REGN3470 and REGN3471) or 3-fold (REGN3479) increase in *k*_d_ values at pH 5.0, indicating that at this pH, binding to GP was still tight. In addition, binding to sGP was measured. Each antibody was captured onto Octet HQX sensors (Fortebio) and immersed in 300 nM solutions of EBOV sGP, EBOV GP, or irrelevant negative control ligand. Whereas all 3 mAbs bound EBOV GP, only REGN3471 demonstrated specific binding to sGP (Table 3), suggesting that it binds within the first 295 amino acids of the common region of both GP and sGP.

**Table 1.** Kinetic Binding Parameters of REGN3470, RENG3471, and REGN3479 on EBOV GP

Antibody	EBOV GP.10xhis Kinetic Binding Parameters			
	*k*_a_ (M^−1^s^−1^)	*k*_d_ (s^−1^)	*K*_D_ (M)	*t*_1/2_ (min)
REGN3470	3.05 × 10^4^	2.36 × 10^–4^	7.74 × 10^–9^	48.9
REGN3471	1.45 × 10^4^	1.22 × 10^–4^	8.42 × 10^–9^	94.7
REGN3479	4.81 × 10^4^	1.43 × 10^–4^	2.97 × 10^–9^	80.8

Abbreviations: EBOV, Ebola virus; GP, glycoprotein.

**Table 2.** Dissociation Kinetics of REGN3470, RENG3471, and REGN3479 from EBOV GP at pH 7.4, pH 6.0, and pH 5.0

Antibody	pH 7.4		pH 6.0		pH 5.0	
	*k*_d_ (1/s)	*t*_1/2_ (min)	*k*_d_ (1/s)	*t*_1/2_ (min)	*k*_d_ (1/s)	*t*_1/2_ (min)
REGN3470	4.32 × 10^–4^	26.7	3.67 × 10^–4^	31.4	4.91 × 10^–4^	23.5
REGN3471	1.95 × 10^–4^	59.1	2.21 × 10^–4^	52.3	3.32 × 10^–4^	34.8
REGN3479	7.83 × 10^–5^	147.6	2.14 × 10^–4^	55.1	2.02 × 10^–4^	57.3

Abbreviations: EBOV, Ebola virus; GP, glycoprotein.

**Table 3.** Binding of REGN3470, REGN3471, and REGN3479 to EBOV GP and EBOV sGP

	Response of 300 nM Protein Binding to mAb		
mAb	EBOV GP.10xHis	EBOV sGP.mmh	Irrelevant mmh Tagged Protein
REGN3470	0.61	0.02	0.01
REGN3471	0.55	0.19	0.01
REGN3479	0.55	0.00	−0.01

Abbreviations: EBOV, Ebola virus; GP, glycoprotein; mAb, monoclonal antibody; sGP, soluble GP.

**Figure 1.**
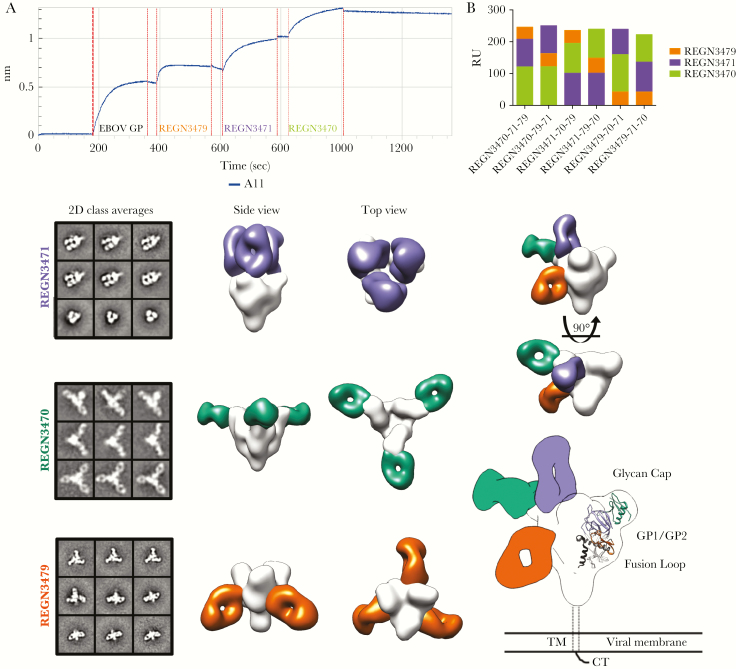
REGN3470, REGN3471, and REGN3479 bind to separate epitopes on the Ebola virus (EBOV) glycoprotein (GP). (A) Sensorgram demonstrating simultaneous binding of all 3 monoclonal antibodies (mAbs) on EBOV GP.10xhis captured on a CM5 sensor chip. Real-time increases in resonance are shown after the sequential addition of EBOV GP.10xhis, followed by REGN3479, REGN3470, and REGN3471 (graph foreshortened at indicated vertical lines to facilitate presentation). (B) Graph showing the maximal Response units (RU) values obtained from the experimental format shown in (A), for all possible orders of addition of the 3 mAbs. (C) Negative-stain electron microscopy of REGN3470, REGN3471, and REGN3479 bound to EBOV GPΔTM. Fragment antigen-binding agents (Fabs) bound to EBOV GPΔTM were examined by single-particle, negative-stain electron microscopy (EM). Far left panels show representative 2-dimensional reference-free class averages of Fab:GP complexes. Central panels show side views (parallel to the viral surface) and top views (perpendicular to the viral surface, down the 3-fold axis of symmetry) of reconstructions of Fabs bound to EBOV GPΔTM (in white), with Fabs segmented and colored purple (top, REGN3471), green (middle, REGN3470), and orange (bottom, REGN3479). Right panels show combined reconstructions of REGN3471, REGN3470, and REGN3479 on a single EBOV GPΔTM, demonstrating the relative locations of the epitopes on GP from the 3 different competition groups. Maps were aligned onto EBOV GPΔTM from the c13C6:c4G7 reconstruction (EMDB 6152). Bottom right, model of the targeted epitopes showing additional detail of the predicted membrane-bound GP.

Two methods were used to demonstrate that the 3 mAbs bind to non-overlapping epitopes on EBOV GP. First, cross-competition between paired mAbs was assessed by using the Octet HTX system. After the capture of EBOV GP protein onto Octet sensors, the first test antibody was allowed to bind to saturation, followed by exposure to the second test antibody. All possible paired combinations of EBOV-specific mAbs and an isotype control were tested (Table 4). This assay format showed that REGN3470, REGN3471, and REGN3479 do not compete for binding to EBOV GP. To substantiate whether these antibodies are able to bind the EBOV GP simultaneously, a 3-step sequential-binding study was performed using SPR. Saturating amounts of each mAb were applied in separate, sequential steps over an EBOV GP sensor chip surface, using all permutations for the order of antibody addition. Figure 1A shows a sensorgram displaying sequential increases in binding signal resulting from injection of EBOV GP to the sensor chip, followed by the injection of REGN3479, REGN3471, and REGN3470. Control experiments demonstrated that repeated injections of the same antibody did not result in additional binding signal, indicating that the binding sites for each antibody were saturated by the first injection (data not shown). Similar results were obtained for all permutations in the order of antibody injections (Figure 1B), demonstrating that REGN3470, REGN3471, and REGN3479 can bind simultaneously to EBOV GP.

**Table 4.** Competitive/Noncompetitive Binding of REGN3470, REGN3471, and REGN3479 to EBOV GP

	Response of 50 μg/mL mAb 2			
	Binding mAb 1-Bound EBOV GP.10xhis (nm)			
mAb	REGN3470	REGN3471	REGN3479	Isotype
REGN3470	0.04	0.48	0.32	0.03
REGN3471	0.41	0.07	0.31	0.02
REGN3479	0.46	0.44	0.08	0
Isotype	0.52	0.5	0.33	0.02

Abbreviations: EBOV, Ebola virus; GP, glycoprotein; mAb, monoclonal antibody.

To further define the antibody binding sites, we used single-particle, negative-stain electron microscopy (EM) of EBOV GP bound to Fabs prepared from each mAb. The images were used to create the reconstructions are shown in Figure 1C. Consistent with the SPR findings, comparison of these reconstructions indicates that each antibody binds to a different site on the GP molecule. Antibody REGN3471 (Figure 1C, purple) has the steepest angle of approach on GP, binding nearly perpendicular to the viral surface. The antibody binds within the chalice structure at or near the head of GP and may partially contact the glycan cap. This placement allows the GP trimer to accommodate 3 REGN3471 Fabs, despite the very tight clustering of the epitope. REGN3470 (Figure 1C, green) binds parallel to the viral surface on the outside of the glycan cap. Finally, REGN3479 (Figure 1C, orange) binds at the base of GP, between protomers of GP1/GP2.

### Functional Characterization of REGN3470, REGN3471, and REGN3479 In Vitro and in Guinea Pigs

The functional properties of each mAb were characterized by determining their in vitro neutralization ability, their in vitro capacity to elicit FcγRIIIa signaling in effector cells, and their ability to block lethal EBOV disease in guinea pigs.

**Figure 2.**
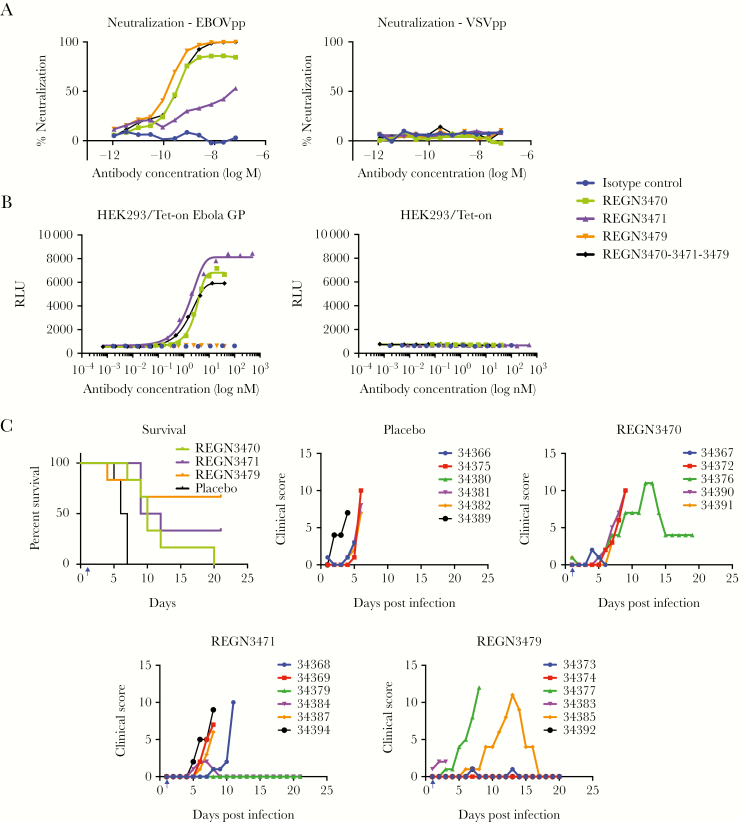
REGN3470, REGN3471, and REGN3479 monoclonal antibodies (mAbs) display different functional properties and protect guinea pigs from lethal Ebola virus (EBOV) disease. (A) Neutralization of virus infection of Huh7 cells challenged with EBOV Zaire 2014 glycoprotein (GP) or vesicular stomatitis virus (VSV) GP-pseudotyped lentiviruses by REGN3470, REGN3471, and REGN3479, alone or in a 1:1:1 cocktail. (B) Activation of FcγRIIIa signaling in engineered Jurkat cells mixed with HEK293 target cells expressing EBOV Zaire 2014 GP in the presence of REGN3470, REGN3471, and REGN3479, alone or in a 1:1:1 cocktail. (C) Survival and clinical scores of guinea pigs infected with 1000 plaque-forming units of guinea pig-adapted EBOV given a single dose of REGN3470, REGN3471, REGN3479, or a control mAb (6 animals per group) at day 1 postinfection.

The ability of REGN3470, REGN3471, and REGN3479 to neutralize lentiviral particles pseudotyped with Makona strain EBOV GP is shown in Figure 2A. REGN3470 and REGN3479 neutralized pseudovirus particles with half maximal inhibitory concentration (IC_50_) values of 0.27 and 0.17 nM, respectively, although neutralization with REGN3470 never exceeded 90%. In contrast, REGN3471 displayed weak neutralization (<50%) and only at high antibody concentrations. As a cocktail, the 3 antibodies demonstrated potent neutralization with an IC_50_ of 0.39 nM. None of the antibodies blocked the entry of VSV-G-pseudotyped control particles, indicating that their activity is specific for EBOV GP (Figure 2A). In addition, we tested the antibodies in a live virus PRNT assay using Makona, Mayinga, guinea pig-adapted Mayinga, and Kikwit EBOV strains (Table 5). REGN3479 was a potent neutralizer of virus infectivity across all strains tested (PRNT-50 value = 0.1–0.2 nM for all strains), whereas REGN3470 and REGN3471 did not display significant neutralization in this assay. The difference in neutralizing potency is consistent with other published data showing that pseudotyped viruses are more easily neutralized than wild-type viruses and probably reflects the lesser amount of GP present on pseudovirions [36].

**Table 5.** In Vitro Neutralization of EBOV Makona, Mayinga, Guinea Pig-Adapted Mayinga, and Makona Strains by REGN3470, RENG3471, REGN3479, and KZ52

Antibody	Makona		Mayinga		Guinea Pig Adapted		Kikwit	
	PRNT-50	PRNT-80	PRNT-50	PRNT-80	PRNT-50	PRNT-80	PRNT-50	PRNT-80
	(nM)	(nM)	(nM)	(nM)	(nM)	(nM)	(nM)	(nM)
REGN3470	NA	NA	NA	NA	NA	NA	NA	NA
REGN3471	NA	NA	NA	NA	NA	NA	NA	NA
REGN3479	0.2	1.2	0.1	0.2	0.2	0.6	0.1	0.3
Isotype Control	NA	NA	NA	NA	NA	NA	NA	NA
KZ52	5.2	x	2	x	1.3	13.3	4.7	x

Abbreviations: EBOV, Ebola virus; NA, no significant neutralization detected; PRNT, plaque reduction neutralization.

Effector functions of antibodies are recognized as important in clearance of many virus types by cell-mediated cytotoxicity. Its importance in clearance of EBOV disease is presently unclear, but previous reports indicated that ADCC is elicited by an antibody isolated from a human survivor [13], and studies have suggested that reduced fucose forms of nonneutralizing mAbs are more efficacious in animal models of EBOV infection than the same mAbs with normal levels of fucose [16]. The ability of REGN3470, REGN3471, REGN3479, or a cocktail of the 3 antibodies to mediate FcγRIIIa signaling in engineered Jurkat cells was evaluated in the presence of HEK293 cells expressing Makona strain EBOV GP (to mimic infected cell targets) or HEK293 parental control cells (Figures 2B). In the presence of EBOV GP-expressing cells, REGN3470 and REGN3471 induced a dose-dependent activation of FcγRIIIa signaling in effector cells with half maximal effective concentration (EC_50_) values of 2.9 and 1.6 nM, respectively. The REGN3470-3471-3479 cocktail (1:1:1 equimolar ratio) also induced FcγRIIIa signaling with an EC_50_ of 1.7 nM. No activation was observed in the presence of control cells. These results indicate that binding of REGN3470 and REGN3471 to EBOV GP-expressing target cells induced avidity-driven Fc binding to FcγRIIIa on effector cells, resulting in activation of signaling. REGN3479 had no activity, indicating that this antibody, although strongly neutralizing, does not activate FcγRIIIa signaling in engineered Jurkat cells, even though it bound to the target cell surface (results not shown).

To determine whether the in vitro properties of each antibody translated into in vivo activity, they were evaluated for their postexposure therapeutic efficacy against a lethal EBOV challenge in the guinea pig model of infection. Guinea pigs were chosen because they have been used to predict therapeutic benefit for antibody-cocktails eventually tested in NHPs [12]. Groups of 6 animals (approximately 400 grams each) were challenged with a target dose of 1000 pfu of guinea pig-adapted EBOV, followed 24 hours later by a single 5-mg dose of antibody or placebo control. Guinea pigs were monitored twice daily for 21 days. Kaplan-Meier survival curves for each cohort and the clinical scores of each animal in each treatment group are shown in Figure 2C. All of the guinea pigs in the placebo group succumbed to EBOV disease by day 7. In contrast, postchallenge administration of each antibody was shown to delay EBOV disease symptom onset and prevent or delay death during the observation period. REGN3479, the most potent neutralizer in vitro, displayed the greatest individual efficacy, with 66% of treated animals surviving. REGN3471 protected approximately 33% of infected animals, whereas, REGN3470 improved median survival by 4 days, but all animals eventually succumbed to disease. The clinical scores indicated delayed onset of disease signs for all mAb-treated animals compared with placebo treatment. In addition, surviving animals in the REGN3471- and REGN3479-treated groups displayed limited clinical signs of infection. This study shows that in this disease model antibodies that only block infection by neutralization (REGN3479), only have effector functions via signaling through FcγRIIIa (REGN3471), or combine both activities (REGN3470) show benefit in vivo when administered as a monotherapy. Taken together, the in vitro and in vivo results demonstrate that each of the 3 mAbs selected has a different binding and functional activity profile that confers protection from disease and lethality in an in vivo model of EBOV disease.

### Therapeutic Efficacy of the REGN3470-3471-3479 Cocktail in Three Nonhuman Primate Studies

**Figure 3.**
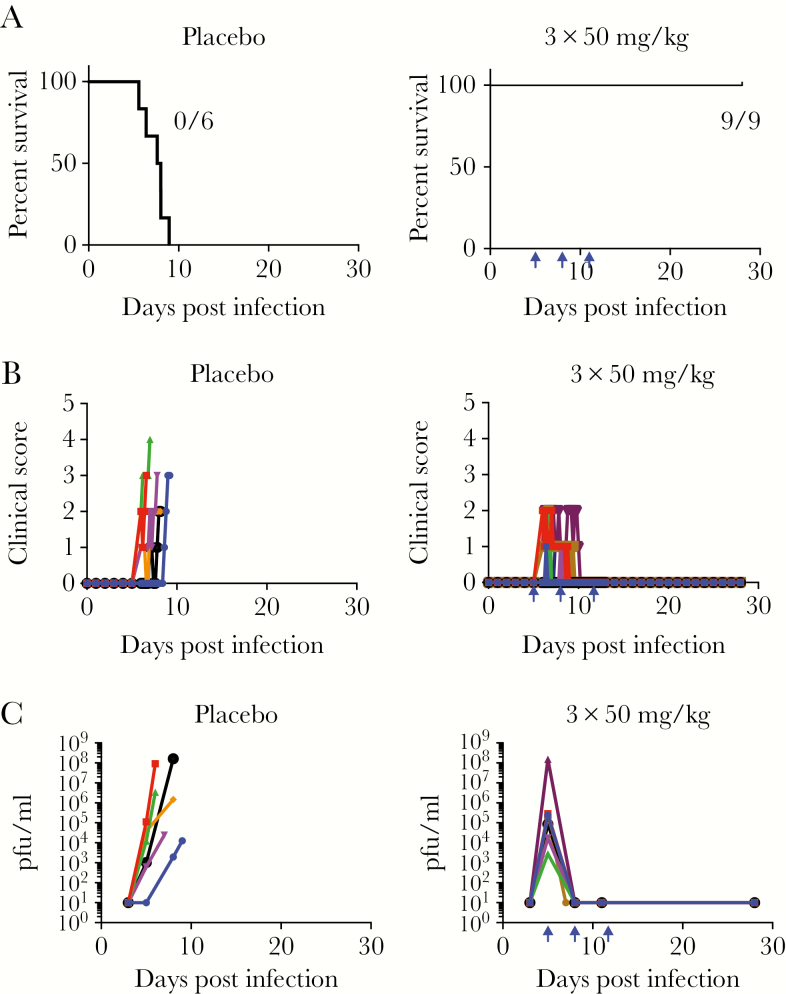
Three doses of REGN3470-3471-3479 cocktail protect rhesus macaques from disease after infection with the Kikwit strain of Ebola virus. (A) Survival plots, (B) signs of clinical disease, and (C) viral load data for animals treated with placebo (6 animals) or with 3 doses of 50 mg/kg (1:1:1) of REGN3470-3471-3479 cocktail on days 5, 8, and 11 postinfection (9 animals).

The rhesus macaque model of EBOV infection recapitulates key features of human disease [37] and was used to demonstrate the therapeutic potential of REGN3470, REGN3471, and REGN3479 in 3 independent studies designed to assess the effects of antibody dose and regimen on efficacy. The first study showed that 3 postinfection doses of the REGN3470-3471-3479 cocktail administered 1:1:1 (50 mg/kg total antibody) resulted in 100% protection from lethal disease. Rhesus macaques were challenged with a target dose of 1000 pfu of the Kikwit strain of EBOV by intramuscular injection and were randomized into 2 groups. Group 1 (control arm) contained 6 animals that were each administered an intravenous placebo control. Group 2 (treatment arm) contained 9 animals that were administered the REGN3470-3471-3479 cocktail intravenously at 50 mg/kg (1:1:1) on days 5, 8, and 11 postinfection. Animals were monitored daily for signs of EBOV disease. As shown in Figure 3A, all 6 placebo-treated animals succumbed to EBOV disease by day 9 postinfection. In contrast, despite showing disease symptoms on day 5, when treatment began, none of the 9 animals that received the antibody cocktail succumbed to disease. Clinical scores (Figure 3B) indicated that disease symptoms in the treated animals were also abrogated and that all treated animals showed no obvious signs of disease before the third dose of the cocktail was administered on day 11. Analysis of serum viral load by plaque assay (Figure 3C) indicated that all treated animals had undetectable circulating replication-competent virus after the first dose of the cocktail. Body temperatures recorded by telemetry (Supplemental Figure 1) indicated that all animals in the placebo group had sustained fever (Supplemental Figure 1A–F, red and purple dots) that persisted until succumbing to disease. Likewise, the antibody cocktail-treated animals exhibited fever beginning on day 3–4 postinfection. However, in contrast to the placebo group, the fever had ended, for most animals, by day 9, providing an additional clinical measure indicating that animals were recovering 4 days after initiation of treatment, before the third dose of the cocktail was administered.

**Figure 4.**
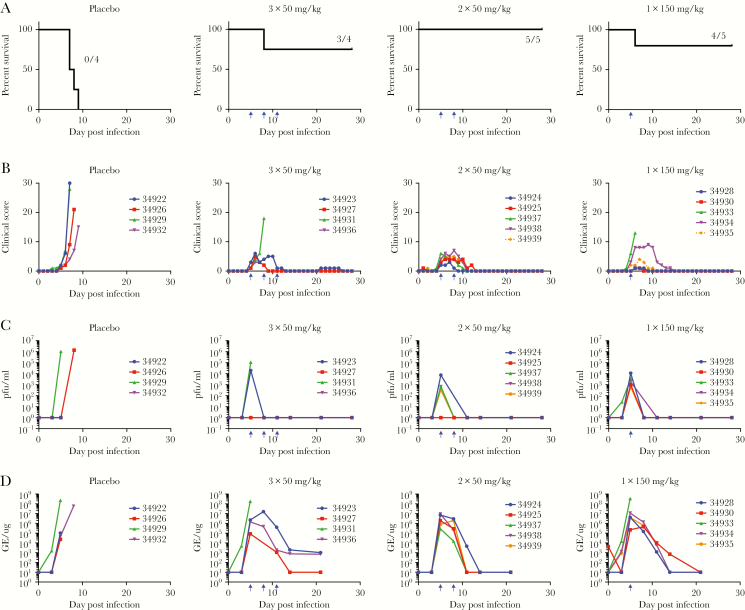
REGN3470-3471-3479 cocktail protects rhesus macaques from disease after infection with the Kikwit strain of Ebola virus using various doses/dosing regimens. (A) Survival, (B) signs of clinical disease, and viral load by (C) plaque assay or (D) genome copies for animals in rhesus macaques treated with placebo (4 animals), 3 doses of 50 mg/kg (1:1:1) of cocktail on days 5, 8, and 11 postinfection (4 animals), 2 doses of 50 mg/kg (1:1:1) of REGN3470-3471-3479 cocktail on days 5 and 8 (5 animals), or 1 dose of 150 mg/kg (1:1:1) of cocktail on day 5 (5 animals).

To evaluate whether single-dose or 2-dose regimens were similarly protective, a second independent postexposure treatment study was performed at a different BSL-4 facility. Eighteen rhesus macaques were challenged with a target dose of 1000 pfu of the Kikwit strain of EBOV and divided into 4 groups that received placebo control (4 animals), 3 doses of 50 mg/kg of the antibody mixture (1:1:1) on days 5, 8, and 11 postinfection (4 animals), 2 doses of 50 mg/kg of the antibody mixture on days 5 and 8 postinfection (5 animals), or 1 dose of 150 mg/kg of the antibody mixture on day 5 postvirus challenge. Animals were monitored daily for mortality and clinical symptoms (Figure 4A and B). As in the previous study, all placebo control-treated animals succumbed to EBOV disease by day 9. In contrast, treatment resulted in survival of most animals in each treatment group, despite the finding that all animals showed multiple clinical signs of disease before treatment initiation (Figure 4B). Only 1 animal succumbed to the infection in the 3 × 50 mg/kg and 1 × 150 mg/kg groups, and all animals survived in the 2 × 50 mg/kg group. There were no significant differences between the treatment arms, indicating that fewer doses (and less total administered antibody) or a single administration of the antibody cocktail provide a similar therapeutic result to what was previously observed with 3 × 50 mg/kg doses of cocktail. Similar to what was observed in the first study, combined clinical scores of placebo and antibody-treated animals (Figure 4B) indicated that all animals had advanced disease that was controlled by the treatment, and the majority of these infected animals displayed no symptoms after approximately day 10 postinfection. Finally, virus load was measured in the sera of infected control and treated animals by 2 different assays: a plaque assay to detect infectious virus (Figure 4C) and quantitative PCR to detect the presence of virus genomes (Figure 4D). These viral load studies confirmed that antibody treatment (but not placebo treatment) controlled EBOV infection, reducing virus loads by >10^6^-fold, and showed that the 2 animals that succumbed to EBOV disease in the treated groups displayed viral genome copy numbers higher than the other members of their treated cohorts. In addition, these data show similar levels of virus load control in all treated animals regardless of treatment regimen.

**Figure 5.**
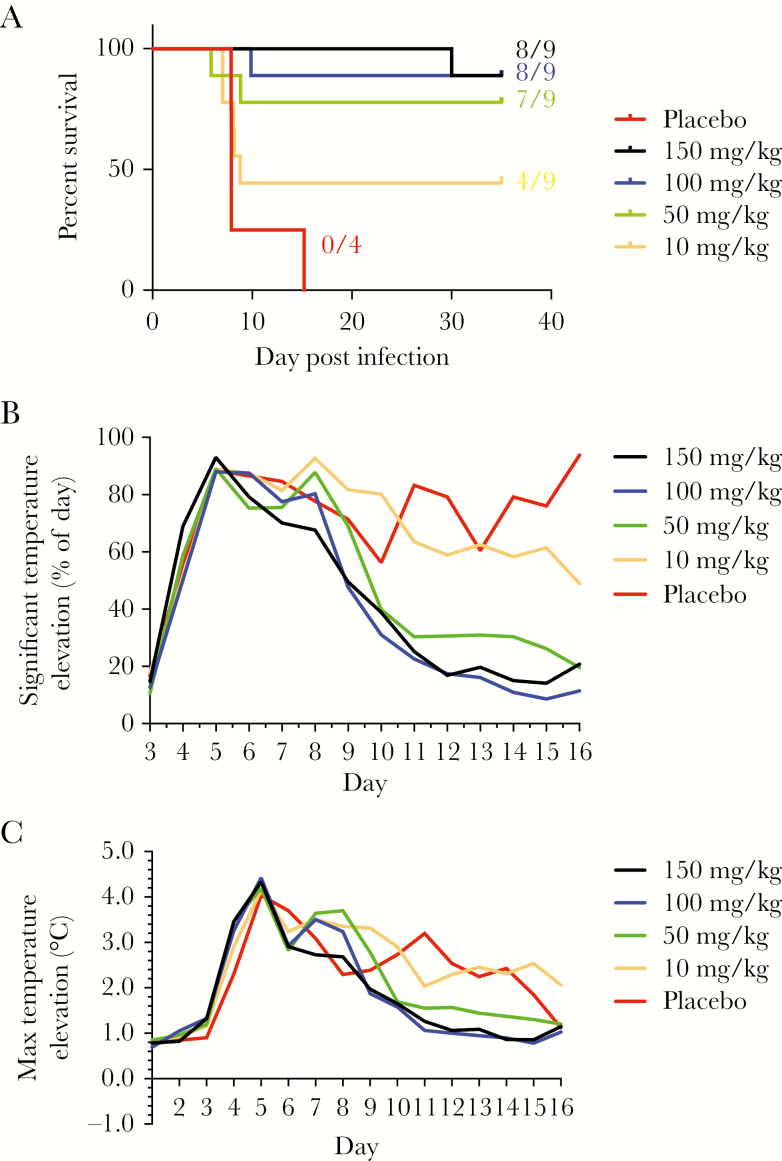
A single dose of REGN3470-3471-3479 cocktail protects rhesus macaques from a lethal infection with the Kikwit strain of Ebola virus. (A) Survival and (B and C) animal temperature data for rhesus macaques treated with placebo (4 animals) or a single dose of 10 mg/kg (9 animals), 50 mg/kg (9 animals), 100 mg/kg (9 animals), or 150 mg/kg (9 animals) (1:1:1) of REGN3470-3471-3479 cocktail on day 5 postinfection.

A third study was designed to test a single treatment dose (for clinical ease of use in an outbreak setting) and evaluate the minimal efficacious dose of the REGN3470-3471-3479 cocktail. Forty rhesus macaques were challenged and divided into 5 groups that received placebo control (4 animals), 150 mg/kg (9 animals), 100 mg/kg (9 animals), 50 mg/kg (9 animals), and 10 mg/kg (9 animals) of the antibody cocktail (1:1:1) on day 5 postinfection. As shown in Figure 5A, all 4 placebo control-treated animals succumbed to EBOV disease. As seen in the second study, despite clear disease signs being present, administration of 150 mg/kg antibody cocktail resulted in almost complete survival of infected animals (8 of 9 animals). In addition, a single treatment of 100 mg/kg resulted in a similar high survival (8 of 9 animals), and a single treatment of the 50 mg/kg led to survival of 7 of 9 animals (nonsignificant difference compared with higher-dose groups), whereas 10 mg/kg had a partial effect, because 5 of 9 succumbed to the infection despite treatment. Duration of fever (Figure 5B) and the extent of temperature increase (Figure 5C) were identical for the 150 mg/kg and 100 mg/kg groups, indicating that these 2 doses of the cocktail had similar effect on controlling fever. In comparison, animals that received the 50 mg/kg dose were slightly impaired at controlling their fever, whereas the 10 mg/kg group displayed temperature readings similar to the placebo-treated group, even though a significant number of animals survived in this cohort. Thus, a single-dose treatment with the REGN3470-3471-3479 cocktail afforded high-level, dose-dependent, postexposure protection against lethal EBOV disease and that 100 mg/kg was the lowest dose tested that gave best control of symptoms.

## DISCUSSION

The catastrophic outbreak of EBOV in West Africa in 2013–2016 and the current outbreak in Congo have emphasized the need to develop rapid, systematic approaches for development of therapeutics for treatment of disease. Multiple classes of EBOV disease therapeutics, ranging from small molecule inhibitors that act on viral replication to mAbs that recognize viral proteins, have been described [38]. Human mAbs are particularly appealing because they display predictable pharmacokinetics and tolerability, and their antipathogen activities can be rapidly evaluated in vitro based on established mechanisms of action. Fully human mAbs with potent antiviral activity can be generated rapidly and reliably using a platform based on humanized mice as demonstrated in the present report and previously for development of mAbs to treat infections with Middle East respiratory syndrome coronavirus (MERS-CoV) [22].

In the work described here, we developed a cocktail of antibodies to (1) combine biological activities and improve efficacy/outcomes, (2) reduce potential for escape and resistance, and (3) increase the potential utility for future outbreaks as virus sequences evolve. To this end, we selected candidate mAbs based on in vitro antibody properties suggested as predictive of in vivo efficacy in previous studies. Both neutralizing and nonneutralizing antibodies have previously been suggested to be efficacious in guinea pig and NHP models, so assays were established to measure neutralization of EBOV pseudotypes, as well as assays to evaluate ability to trigger FcγRIIIa signaling, and binding to sGP (which likely plays a role in EBOV pathogenesis).

Using this approach, we generated a pool of antibodies to EBOV GP and selected 3 leads that had the following activities: (1) REGN3470 neutralizes EBOV GP pseudotypes, triggers FcγRIIIa signaling, but does not bind sGP; (2) REGN3471 has no neutralizing activity, triggers high levels of FcγRIIIa signaling, but does bind sGP; and (3) REGN3479 potently neutralizes EBOV GP pseudotypes and live EBOV, but does not trigger FcγRIIIa signaling or bind sGP. The 3 antibodies were purposely chosen to bind distinct epitopes in the GP to hinder development of viral resistance. After the rapid screening and selection of these mAbs, the guinea pig model was used to validate them. An antibody with FcγRIIIa signaling but no neutralizing activity (REGN3471), a novel single mAb that displayed both neutralizing and FcγRIIIa signaling activity (REGN3470), or a mAb with high levels of neutralizing activity (REGN3479) can delay or prevent death in this model. In this case, the strongly neutralizing antibody appeared more protective than the other 2 antibodies. However, the human Fc may not properly trigger guinea pig Fcγ receptors, so it is hard to interpret the contribution of effector activity in this model.

Analyses performed using single-particle, negative-stain EM provided a wealth of information on the binding sites for the 3 mAbs. These studies revealed that REGN3471 has the steepest angle of approach on EBOV GP, binding perpendicular to the viral surface at an angle of approximately 90 degrees. The EBOV GP trimer was able to accommodate 3 REGN3471 Fabs, despite the tight clustering of the epitope within the chalice structure of the trimer at or near the head of GP, in an area that likely overlaps with the glycan cap. REGN3471 appears to bind even closer to the center of the chalice than does mAb c13C6 (that forms part of the ZMapp cocktail) [31]. Compared with mAb114, a neutralizing human mAb obtained from a long-term human survivor of EBOV disease [39], REGN3471 binds further away from the NPC1 Loop C binding site, which may explain why this antibody is not neutralizing [39]. REGN3470 binds the EBOV GP parallel to the viral surface on the outside of the glycan cap, a position that could help to explain neutralization and its ability to trigger FcγRIIIa. Finally, REGN3479 binds at the base of GP, between protomers of GP1/GP2. The epitope lies near the internal fusion loop and cathepsin cleavage site, similar to the neutralizing antibodies derived from a long-term survivor, which were recently crystalized [39]. The angle of REGN3479 is rotated 90 degrees compared with mAb100, and the angle of approach is slightly upward from the viral surface. Based on its binding site, the potent neutralization activity of REGN3479 may be due to blockade of cathepsin cleavage or release of the fusion loop upon receptor binding, similar to the mechanism proposed for mAb100 [39].

More importantly, the functional characterization and structural data also demonstrate that the REGN3470-3471-3479 cocktail of VelocImmune mAbs reflects the diversity of mAbs produced in wild-type mice (such as 13C6, 2G4, 4G7) [40, 41] and mAbs obtained from survivors of EBOV disease (such as KZ52, mAb114, and mAb100) [39, 42].

To further assess whether REGN3470, REGN3471, and REGN3479 are appropriate candidates for treatment of EBOV disease in humans, they were evaluated in the rhesus macaque model of disease. This work was performed using a clinical (nonadapted) EBOV isolate that replicates many of the key pathophysiological aspects to EBOV infection in man [37]. Furthermore, the antibody mixture used was the same lot of clinical-grade material that is undergoing human testing (clinicaltrials.gov number NCT02777151). Using this model, 3 different studies were performed and confirmed the therapeutic benefit of the REGN3470-3471-3479 cocktail. More importantly, in postexposure studies where treatment was initiated after the appearance of clinical signs of disease, in 2 separate studies conducted at 2 different BSL-4 laboratories, 2 doses of 50 mg/kg and a single infusion of 100 mg/kg or 150 mg/kg of REGN3770-3471-3479 prevented death in >88% of the animals.

## CONCLUSIONS

In summary, we used VelocImmune mice to generate a cocktail of 3 fully human antibodies to EBOV GP that provide complementary mechanisms of actions, reflect the diversity of antibodies isolated by conventional methods, incorporate novel specificities, and demonstrate high-level postexposure protection from lethal EBOV disease in NHPs. The entire process of antibody generation, selection, preclinical evaluation, and readiness for human testing was completed in <1 year, and the cocktail is currently being tested in healthy volunteers in preparation for future EBOV outbreaks. More importantly, this rapid isolation of fully human mAbs previously reported for MERS-CoV [22] and here for EBOV demonstrate the usefulness of a combined platform of technologies, which is currently being applied to Zika virus, and it may also be applicable to future emerging viral threats.

## Supplementary Data

Supplementary materials are available at *The Journal of Infectious Diseases* online. Consisting of data provided by the authors to benefit the reader, the posted materials are not copyedited and are the sole responsibility of the authors, so questions or comments should be addressed to the corresponding author.

Supp Fig1

## Notes

***Acknowledgments.*** Regeneron authors thank the Preclinical Manufacturing and Process Development group for rapid scale up of antibodies, Sumathi Sivapalasingam and Laura Pologe for their input and guidance, and Yasmin Khan, Emily Barrett, Jordan Zhu, and Kalyani Penta for program management. The US Army Medical Research Institute of Infectious Diseases (USAMRIID) thanks Adrienne Kimmel, John Braun, Tom Sprague, and Kenny Lin for drug preparation and data entry, Lori Eccleston, Susan Newcomb, and Carly Wlazlowski for data integrity, the USAMRIID polymerase chain reaction core for circulating genomes data, Dennis Taylor for project management, and Beverly Bassett for technical writing assistance

***Financial support.*** This work was funded in part with Federal funds from the Department of Health and Human Services; Office of the Assistant Secretary for Preparedness and Response; and Biomedical Advanced Research and Development Authority (BARDA), under Contract No. “HHSO100201500013C”. Funding from BARDA was also used for animal studies performed at USAMRIID and Texas Biomedical Research Institute. Funding was provided by Regeneron Pharmaceuticals, Inc.

***Potential conflict of interests.*** K. E. P., D. D., A. B. W., J. F., M. T., A. R., Y. Y., A. B., K. Y., T. P., D. B., R. B., G. C., J. M., T. T. H., L. L., P. M., W. O., N. S., and C. A. K. are employees of Regeneron Pharmaceuticals, Inc. All authors have submitted the ICMJE Form for Disclosure of Potential Conflicts of Interest. Conflicts that the editors consider relevant to the content of the manuscript have been disclosed.
